# CSF sTREM2: marking the tipping point between preclinical AD and dementia?

**DOI:** 10.15252/emmm.201606245

**Published:** 2016-03-14

**Authors:** Suzanne E Schindler, David M Holtzman

**Affiliations:** ^1^ Department of NeurologyWashington University School of MedicineSt. LouisMOUSA; ^2^ Knight Alzheimer's Disease Research CenterWashington University School of MedicineSt. LouisMOUSA; ^3^ Hope Center for Neurological DisordersWashington University School of MedicineSt. LouisMOUSA

**Keywords:** Biomarkers & Diagnostic Imaging, Neuroscience

## Abstract

Biomarkers for Alzheimer's disease (AD) have improved our understanding of the temporal sequence of biological events that lead to AD dementia (Jack *et al*, 2013). AD is characterized neuropathologically by amyloid plaques comprised of the amyloid‐β peptide and neurofibrillary tangles comprised of tau. Brain amyloid deposition, as evidenced by a decline in amyloid‐β peptide 42 (Aβ42) in the cerebrospinal fluid (CSF) or by binding of amyloid PET ligands, is thought to be a key initiating event in AD and begins many years prior to the onset of dementia. A rise in CSF tau and phosphorylated tau in the setting of Aβ deposition appears to reflect neurodegeneration and also begins years prior to the onset of dementia but after Aβ deposition has begun to accumulate. Individuals with “preclinical AD,” that is, normal cognition but abnormal AD biomarkers, have a much higher risk for developing AD dementia but may remain cognitively normal for years (Vos *et al*, 2013). While deposition of amyloid and formation of tau tangles are necessary for AD to occur, it is likely that additional events involving inflammation or other processes contribute to crossing the tipping point from preclinical AD to AD dementia. Current efforts are aimed at defining the biomarker(s) that best predict the transition from cognitive normality to abnormality. A biomarker that is closely associated with the onset of cognitive decline could help us to understand the biological events that connect amyloid deposition and tangle formation to cognitive decline and could have significant practical value in AD diagnosis and clinical trial design.

AD researchers have long suspected that inflammation plays an important role in the biology of AD, but genetic evidence was largely lacking until 2013, when two studies reported that rare, missense mutations in TREM2 (triggering receptor expressed on myeloid cells 2) increased risk for AD by about twofold to fourfold (Guerreiro *et al*, 2013; Jonsson *et al*, 2013). Since then the biology of TREM2, and the role of TREM2 in AD, has been a very active area of investigation (for review see Ulrich & Holtzman, 2016). TREM2 is a receptor that is expressed on phagocytic cells, including microglia, and can activate a number of different signaling cascades that modulate inflammatory cytokines and other processes. Mice that express mutant human APP and develop amyloid deposition have upregulation in TREM2 in regions with amyloid deposition (Frank *et al*, 2008). One report suggested that loss‐of‐function mutations in TREM2 decrease the phagocytic activity of microglia and reduce Aβ clearance (Kleinberger *et al*, 2014). Further, mice with brain amyloid deposition and TREM2 haploinsufficiency have decreased clustering of microglia around amyloid plaques (Ulrich *et al*, 2014). Although the details of how TREM2 modulates risk for AD remain to be clarified, it appears that TREM2 helps to mediate a protective inflammatory response to amyloid pathology. Thus, TREM2 may be a link between amyloid pathology and complex downstream events including inflammation.

In their paper, Suárez‐Calvet *et al* (2016) examined CSF levels of the soluble ectodomain of TREM2 (sTREM2) in individuals representing the spectrum of AD, from cognitively normal with no biomarker evidence of AD pathology to preclinical AD to mild cognitive impairment (MCI) to AD dementia. Interestingly, they found that levels of CSF sTREM2 were lowest in controls and preclinical AD, peaked in MCI‐AD, and then declined slightly in AD dementia. The authors also found correlations between CSF sTREM2 and CSF Aβ42, tau, and ptau. CSF sTREM2 levels correlated better with CSF tau and ptau levels than Aβ42 levels, suggesting that elevated sTREM2 levels occur later in the course of the AD process. Two other groups also recently reported that CSF sTREM2 levels are elevated in AD cases compared to cognitively normal controls and that sTREM2 levels are correlated with CSF tau and ptau (Heslegrave *et al*, 2016; Piccio *et al*, 2016). However, these groups did not examine how sTREM2 levels changed across the AD spectrum. Although the findings of Suárez‐Calvet *et al* (2016) are cross‐sectional in nature and must be replicated in cohorts sampled and followed longitudinally, they show that CSF sTREM2 levels may increase at the onset of cognitive decline (see Fig 1).

**Figure 1:**
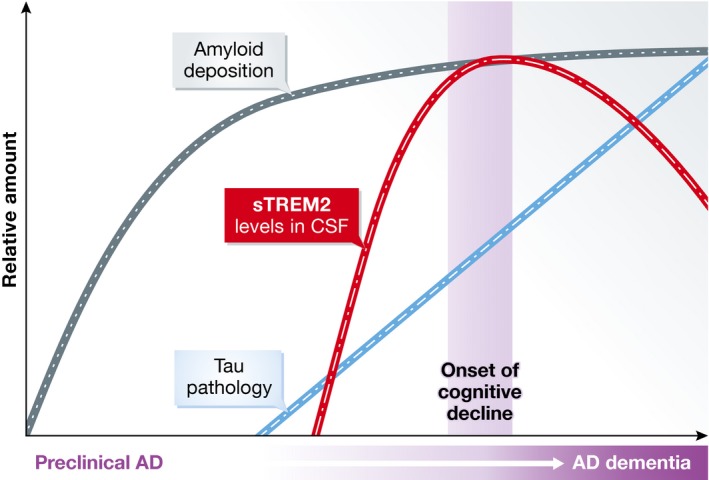
Possible temporal ordering of biomarkers along the AD time course incorporating CSF sTREM2

If CSF sTREM2 levels are reproducibly associated with the onset of cognitive decline, this biomarker could have significant value in AD diagnostics and drug trials. Current clinical CSF diagnostics evaluate levels of Aβ42, tau, and ptau. The combination of low CSF Aβ42 and high tau/ptau is consistent with the presence of AD pathology, but it is not specific—it may occur either in patients with dementia due to AD or preclinical AD. Addition of sTREM2 to CSF testing may improve our ability to determine whether mild cognitive problems are due to AD or other etiologies. CSF sTREM2 levels could also be helpful in AD drug trials. Biomarkers are being used as endpoints as well as enrollment criteria in some clinical trials. If CSF sTREM2 is reliably associated with the onset of cognitive decline, reduced sTREM2 levels in drug‐treated individuals with preclinical AD could indicate a favorable drug effect. Additionally, drugs for AD may be most effective when given at a particular stage of the AD process. Individuals who have abnormal biomarkers for amyloid, tau/ptau but normal CSF sTREM2 may be at an earlier stage of the AD process than individuals with abnormalities in all three biomarkers. If CSF sTREM2 further refines our ability to stage AD, this would allow us to evaluate which drugs are most appropriate for patients at various stages of the AD process. If additional studies confirm the results of Suárez‐Calvet *et al* (2016), CSF sTREM2 levels may be an important biomarker that represents some of the biological events that connect amyloid deposition and neurofibrillary tangle formation to cognitive decline. A biomarker that marks the transition from preclinical AD to dementia could have a major impact on our ability to understand, diagnose, and treat this formidable disease.

## References

[emmm201606245-bib-0001] Frank S , Burbach GJ , Bonin M , Walter M , Streit W , Bechmann I , Deller T (2008) TREM2 is upregulated in amyloid plaque‐associated microglia in aged APP23 transgenic mice. Glia 56: 1438–1447 18551625 10.1002/glia.20710

[emmm201606245-bib-0002] Guerreiro R , Wojtas A , Bras J , Carrasquillo M , Rogaeva E , Majounie E , Cruchaga C , Sassi C , Kauwe JS , Younkin S *et al* (2013) TREM2 variants in Alzheimer's disease. N Eng J Med 368: 117–127 10.1056/NEJMoa1211851 PMC3631573 23150934

[emmm201606245-bib-0003] Heslegrave A , Heywood W , Paterson R , Magdalinou N , Svensson J , Johansson P , Ohrfelt A , Blennow K , Hardy J , Schott J *et al* (2016) Increased cerebrospinal fluid soluble TREM2 concentration in Alzheimer's disease. Mol Neurodegener 11: 3 26754172 10.1186/s13024-016-0071-x PMC4709982

[emmm201606245-bib-0004] Jack CR Jr , Knopman DS , Jagust WJ , Petersen RC , Weiner MW , Aisen PS , Shaw LM , Vemuri P , Wiste HJ , Weigand SD *et al* (2013) Tracking pathophysiological processes in Alzheimer's disease: an updated hypothetical model of dynamic biomarkers. Lancet Neurol 12: 207–216 23332364 10.1016/S1474-4422(12)70291-0 PMC3622225

[emmm201606245-bib-0005] Jonsson T , Stefansson H , Steinberg S , Jonsdottir I , Jonsson PV , Snaedal J , Bjornsson S , Huttenlocher J , Levey AI , Lah JJ *et al* (2013) Variant of TREM2 associated with the risk of Alzheimer's disease. N Eng J Med 368: 107–116 10.1056/NEJMoa1211103 PMC3677583 23150908

[emmm201606245-bib-0006] Kleinberger G , Yamanishi Y , Suarez‐Calvet M , Czirr E , Lohmann E , Cuyvers E , Struyfs H , Pettkus N , Wenninger‐Weinzierl A , Mazaheri F *et al* (2014) TREM2 mutations implicated in neurodegeneration impair cell surface transport and phagocytosis. Sci Transl Med 6: 243ra286. 10.1126/scitranslmed.3009093 24990881

[emmm201606245-bib-0007] Piccio L , Deming Y , Del‐Aguila JL , Ghezzi L , Holtzman DM , Fagan AM , Fenoglio C , Galimberti D , Borroni B , Cruchaga C (2016) Cerebrospinal fluid soluble TREM2 is higher in Alzheimer's disease and associated with mutation status. Acta Neuropathol doi:10.1007/s00401‐016‐1533‐5 10.1007/s00401-016-1533-5 PMC4867123 26754641

[emmm201606245-bib-0008] Suárez‐Calvet M , Kleinberger G , Araque Caballero MA , Brendel M , Rominger A , Alcolea D , Fortea J , Lleó A , Blesa R , Gispert JD *et al* (2016) sTREM2 cerebrospinal fluid levels are a potential biomarker for microglia activity in early‐stage Alzheimer's disease and associate with neuronal injury markers. EMBO Mol Med. 8: 466–476 26941262 10.15252/emmm.201506123 PMC5120370

[emmm201606245-bib-0009] Ulrich JD , Finn MB , Wang Y , Shen A , Mahan TE , Jiang H , Stewart FR , Piccio L , Colonna M , Holtzman DM (2014) Altered microglial response to Abeta plaques in APPPS1‐21 mice heterozygous for TREM2. Mol Neurodegener 9: 20 24893973 10.1186/1750-1326-9-20 PMC4049806

[emmm201606245-bib-0010] Ulrich JD , Holtzman DM (2016) TREM2 function in Alzheimer's disease and neurodegeneration. ACS Chem Neurosci. doi:10.1021/acschemneuro.5b00313 10.1021/acschemneuro.5b00313 26854967

[emmm201606245-bib-0011] Vos SJ , Xiong C , Visser PJ , Jasielec MS , Hassenstab J , Grant EA , Cairns NJ , Morris JC , Holtzman DM , Fagan AM (2013) Preclinical Alzheimer's disease and its outcome: a longitudinal cohort study. Lancet Neurol 12: 957–965 24012374 10.1016/S1474-4422(13)70194-7 PMC3904678

